# Advantages and Limits of Metagenomic Assembly and Binning of a Giant Virus

**DOI:** 10.1128/mSystems.00048-20

**Published:** 2020-06-23

**Authors:** Frederik Schulz, Julien Andreani, Rania Francis, Hadjer Boudjemaa, Jacques Yaacoub Bou Khalil, Janey Lee, Bernard La Scola, Tanja Woyke

**Affiliations:** a DOE Joint Genome Institute, Berkeley, California, USA; b Aix-Marseille Université, IRD, APHM, MEPHI, IHU Méditerranée Infection, Marseille, France; c Department of Biology, Hassiba Ben Bouali University Chlef, Chlef, Algeria; Princeton University

**Keywords:** giant viruses, metagenomics, NCLDV

## Abstract

Giant viruses have large genomes, often within the size range of cellular organisms. This distinguishes them from most other viruses and demands additional effort for the successful recovery of their genomes from environmental sequence data. Here, we tested the performance of genome-resolved metagenomics on a recently isolated giant virus, Fadolivirus, by spiking it into an environmental sample from which two other giant viruses were isolated. At high spike-in levels, metagenome assembly and binning led to the successful genomic recovery of Fadolivirus from the sample. A complementary survey of the major capsid protein indicated the presence of other giant viruses in the sample matrix but did not detect the two isolated from this sample. Our results indicate that genome-resolved metagenomics is a valid approach for the recovery of near-complete giant virus genomes given that sufficient clonal particles are present. However, our data also underline that a vast majority of giant viruses remain currently undetected, even in an era of terabase-scale metagenomics.

**IMPORTANCE** The discovery of large and giant nucleocytoplasmic large DNA viruses (NCLDV) with genomes in the megabase range and equipped with a wide variety of features typically associated with cellular organisms was one of the most unexpected, intriguing, and spectacular breakthroughs in virology. Recent studies suggest that these viruses are highly abundant in the oceans, freshwater, and soil, impact the biology and ecology of their eukaryotic hosts, and ultimately affect global nutrient cycles. Genome-resolved metagenomics is becoming an increasingly popular tool to assess the diversity and coding potential of giant viruses, but this approach is currently lacking validation.

## Abstract (precis)

The discovery of large and giant nucleocytoplasmic large DNA viruses (NCLDV) with genomes in the megabase range and equipped with a wide variety of features typically associated with cellular organisms was one of the most unexpected, intriguing, and spectacular breakthroughs in virology. Recent studies suggest that these viruses are highly abundant in the oceans, freshwater, and soil, impact the biology and ecology of their eukaryotic hosts, and ultimately affect global nutrient cycles. Genome-resolved metagenomics is becoming an increasingly popular tool to assess the diversity and coding potential of giant viruses, but this approach is currently lacking validation.

## INTRODUCTION

Substantial advances in metagenomics and single-cell genomics have rapidly expanded known biodiversity by recovering the sequences of hundreds of thousands of uncultured bacteria and archaea from the environment and from the human microbiome (1–4). Metagenomics has also recently proven to be a powerful method for assessing the diversity and coding potential of environmental viruses (5, 6). Most viral genomes are small, and when found in metagenomic data, they are readily present on a single contig and thus often considered complete or nearly complete (7). However, this is in stark contrast to genomes of large and giant viruses of the nucleocytoplasmic large DNA viruses (NCLDV), which can be up to several megabases (8, 9). Importantly, recent studies showed that these viruses are among the most diverse and abundant entities in marine systems (10, 11) and are also found in a wide range of nonmarine ecosystems (12–17).

Considering the wealth of existing metagenomic data (18), there was a recent surge in studies describing the recovery of giant virus sequences (13, 14, 16, 19–23). Metagenomic discoveries have preceded the physical isolation of some giant viruses, such as the initial reconstruction of Klosneuvirinae genomes from metagenomic sequences (14) with subsequent physical isolation of additional members of this proposed viral subfamily, namely, Bodo saltans virus (24), Yasminevirus (25), and Fadolivirus (26). The genomes from the uncultivated Klosneuvirinae revealed that they encoded comprehensive translation system components (14), subsequently found in isolated tupanviruses (27). Taken together, these studies indicate that metagenomics is of profound value in deriving genomes of giant viruses from the environment, enabling important novel insights into their predicted biology, ecology, and evolutionary history.

We conducted a benchmarking experiment to address whether genome-resolved metagenomics provides a valid approach for the recovery of giant virus genomes from environmental sequence data. Spiking viral particles into a wastewater sample, we tested the performance of commonly used assembly and binning tools, as well as the ability to detect giant virus genomic information in metagenomes.

## RESULTS

**FIG 1:**
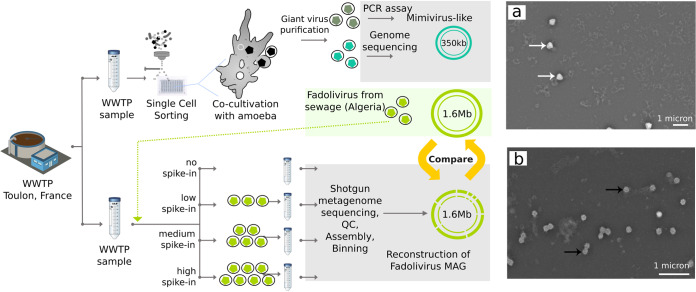
(Left) Benchmarking approach to giant virus metagenomics. Three giant viruses were isolated from wastewater samples by cocultivation with amoebae; Mimivirus-like particles (dark green), Phoenician Marseillevirus (turquoise), and Fadolivirus (light green) are shown. Giant virus particles were identified using a specific PCR assay (Mimivirus-like particles) or using whole-genome sequencing (Fadolivirus, Phoenician Marseillevirus). Fadolivirus particles were purified and spiked into the initial sample at different concentrations (low, medium, and high; see Materials and Methods for more details). Samples with and without viral spike-in were subjected to shotgun metagenome sequencing, quality control (QC), assembly, and binning. The Fadolivirus metagenome assembled genome (MAG) was then compared to the Fadolivirus reference genome. (a and b) Scanning electron micrographs of isolated giant virus obtained with the TM4000 Plus tabletop microscope. (a) Mimivirus-like particles (white arrows). (b) Phoenician Marseillevirus particles (black arrows). Scale bars are indicated on each micrograph.

For giant virus cocultivation experiments, a sample of wastewater was collected from a treatment plant in Toulon, France, and particles within the sample were sorted by flow cytometry into microplates containing host cells. Cocultures were monitored by high content screening (see Materials and Methods for more details), revealing 10 positive wells on Acanthamoeba castellanii strain Neff, while no positive cultures were observed on Vermamoeba vermiformis. Giant virus identification by flow cytometry characteristics showed 2 different populations; the first population corresponded to Mimivirus, and the second population was unidentified. Scanning electron microscopy showed that 6 wells contained typical Mimivirus-like particles (Fig. 1a), and 4 wells contained particles that were 200 to 320 nm in size and resembled Marseillevirus (Fig. 1b). The identity of Mimivirus was validated using a specific PCR assay. The genome of the Marseillevirus-like isolate was sequenced, and phylogenetic analysis of its DNA polymerase gene confirmed this virus as a new member of the *Marseilleviridae*. We named this virus Phoenician Marseillevirus.

**FIG 2:**
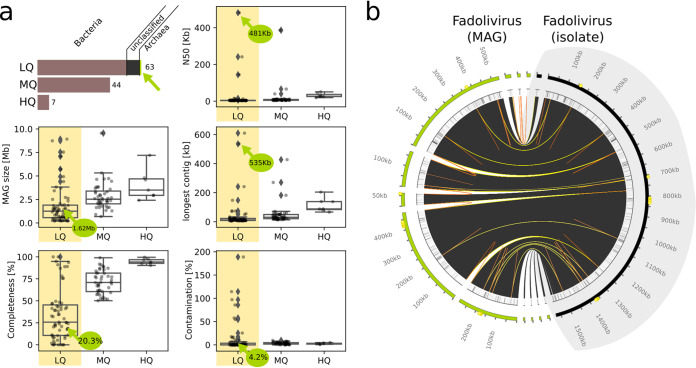
Metagenomic assembly and binning to generate the Fadolivirus metagenome assembled genome (MAG). (a) Bars indicate the total number of low-quality (LQ), medium-quality (MQ), and high-quality (HQ) MAG, as defined by MIMAG standards, after differential coverage binning of the metagenome assembly derived from the sample with the highest virus spike-in. Colors indicate domain-level taxonomic assignment of MAG according to CheckM. Boxplots show different assembly metrics for MAG. Center lines of box plots represent the median, bounds of boxes represent the lower and upper quartile, and whiskers extend to points that lie within the 1.5 interquartile range of the lower and upper quartile. Green arrows indicate the Fadolivirus MAG. (b) Whole-genome synteny plot of the Fadolivirus MAG (light green) compared to the Fadolivirus reference assembly (black). Areas with >99% alignment identity between the two assemblies are highlighted in dark gray. For each assembly, high-identity structural repeats (>95% nucleic acid similarity) with a length of 80 to 200 bp are connected to each other with orange links. Yellow links connect the repeats between both assemblies.

For our metagenomics benchmarking experiment, we began by spiking a portion of the wastewater sample with a known virus, the recently isolated Fadolivirus (26). This viral isolate has a genome size of 1.595 Mb and represents a close relative of Klosneuvirus in the proposed viral subfamily *Klosneuvirinae* (14). Samples were spiked with Fadolivirus at the following levels: no (0 viral particles/ml), low (10^3^ viral particles/ml), medium (10^5^ viral particles/ml), or high (10^7^ viral particles/ml); DNA from each sample was sequenced at the DOE Joint Genome Institute. Metagenomics analysis was then performed using a pipeline routinely used for environmental samples, relying on standard quality control (QC) analysis steps and metaSPAdes (28) assembly (Fig. 1). Binning was performed with MetaBAT 2 (29) using differential coverage, which led to recovery of 115 metagenome assembled genomes (MAG). CheckM-based taxonomic classification (30) assigned 105 MAG a bacterial and 1 MAG an archaeal origin, while 9 MAG remained unclassified due to the absence of phylogenetic marker genes (Fig. 2a). According to the minimum information about a single amplified genome and a metagenome assembled genome (MIMAG) standards (31), 7 of the MAG were of high, 44 of medium, and 64 of low quality (Fig. 2a). The MAG which was predicted to be of archaeal origin (20.3% estimated level of completeness, 4.2% estimated level of contamination; Fig. 2a) comprised viral contigs which represented 99.7% of the Fadolivirus reference genome, and it did not contain any archaeal sequences. This viral MAG was only recovered in the metagenome sample with the high level of viral particle spike-in. To assess the performance of additional commonly used approaches, we also tested metagenomic binning with MetaBAT 2 (29) without differential coverage, MaxBin 2 (32), CONCOCT (33), and DAS_Tool (34) and recovered between 98.3% and 99.7% of the Fadolivirus reference genome (Table 1). However, CONCOCT and MaxBin 2 wrongly assigned several contigs to the Fadolivirus MAG that could not be aligned to the reference genome (Table 1), and the Fadolivirus MAG did not pass the filtering threshold of DAS_Tool, as it lacked most cellular marker genes.

**TABLE 1:** Assembly metrics of the Fadolivirus metagenome assembled genome (MAG) compared to the Fadolivirus reference assembly^*a*^

Parameter	MetaBAT 2-dc^*b*^	MetaBAT 2	CONCOCT	MaxBin 2
Bin size (bp) (Fadolivirus MAG)	1,623,616	1,583,180	1,941,890	1,712,889
Total aligned length (bp)	1,590,159	1,567,605	1,590,159	1,590,159
Unaligned length (bp)	33,031	15,575	351,731	122,730
Genome fraction (%)	99.707	98.297	99.707	99.707
*N*_50_ (bp)	481,715	481,715	481,715	481,715
No. of contigs	12	8	31	21
Largest contig (bp)	535,783	535,783	535,783	535,783
No. of misassemblies	0	0	0	0
No. of aligned contigs	11	7	11	11
No. of unaligned contigs	1	1	20	4
Duplication ratio	1.001	1.001	1.001	1.001
No. of N’s per 100 kb	0	0	0	0
No. of mismatches per 100 kb	16.61	14.87	16.33	14.88
No. of indels per 100 kb	1.64	1.66	1.64	1.64

a MAG from 4 different binning methods are compared. N, unidentified nucleotide.

b MetaBAT 2-dc, MetaBAT 2-differential coverage binning.

The MAG most similar to the genome of Fadolivirus was derived with differential coverage binning in MetaBAT 2 and had a size of 1.623 Mb and an aligned fraction of 99.7% (Fig. 2a; Table 1). It had an *N*_50_ value of 481 kb and comprised 12 contigs, each with a size of at least 5 kb and the largest with a size of 535 kb (Fig. 2a; Table 1). In the viral MAG, 5 kb of the Fadolivirus reference genome were missing. However, the MAG included one additional contig which was not present in the reference genome and two contigs which could only be partially aligned to the reference, totaling 33 kb of unaligned sequence data (Table 1; Fig. 2b). Detailed genome comparison of the aligned fraction between the Fadolivirus isolate and MAG did not identify any misassembled regions and revealed 16 mismatches per 100 kb (Table 1). Furthermore, we evaluated the presence of highly identical repetitive sequences within the Fadolivirus genome and found that such sequences were located at the ends of 8 out of 12 contigs of the metagenome assembly (Fig. 2b).

**FIG 3:**
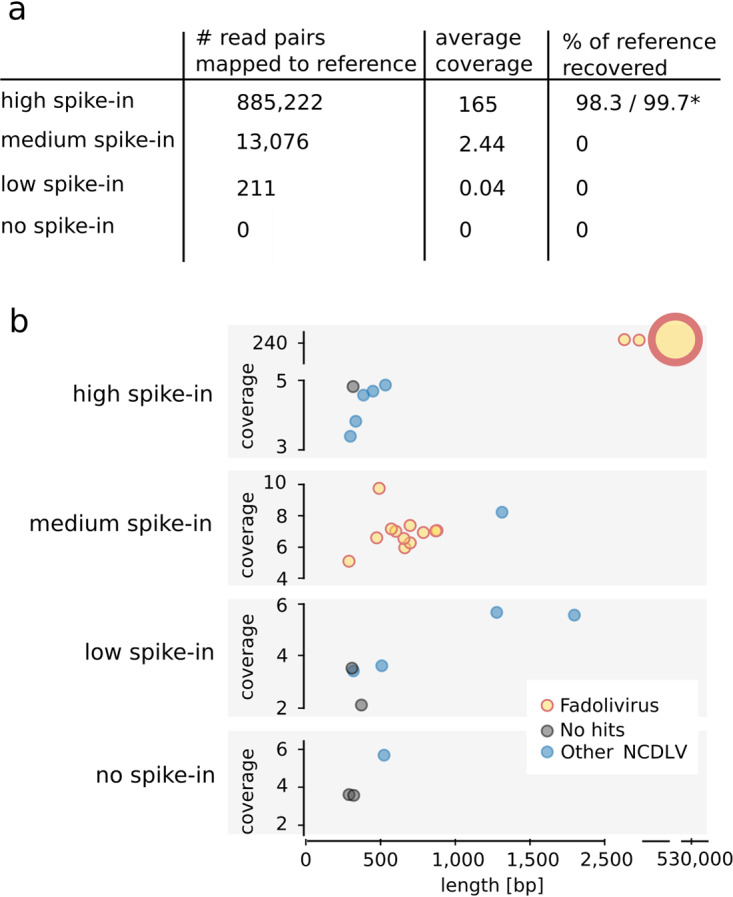
Detection of giant viruses in metagenomic data. (a) Mapping of metagenomic reads from samples with and without viral spike-in to the Fadolivirus reference genome; 98.3% and 99.7% of the Fadolivirus genome could be reconstructed in the metagenome with the highest virus spike-in using MetaBAT 2 (29) and differential coverage binning (*), respectively. (b) Presence of contigs which contained the giant virus MCP gene in samples with and without viral spike-in. Contigs are shown as filled circles which are colored based on the taxonomic origin of the MCP gene. Circle diameter correlates with the total number of MCP genes present on the respective contig. Each contig contained only one MCP gene, with the exception of a single contig in the sample with the high viral spike-in which contained 4 copies of the MCP gene.

To test the detection limit of the spiked-in Fadolivirus and the isolated Phoenician Marseillevirus, we mapped metagenomic reads from each sample to their genomes. In the case of Fadolivirus, the high spike-in samples yielded 68-fold more mapped reads than the medium spike-in and 4,194-fold more mapped reads than the low spike-in (Fig. 3a). Metagenomes from the original samples, i.e., those without Fadolivirus spiked in, did not produce any reads that mapped to its genome. None of the samples contained reads that mapped to the isolated Phoenician Marseillevirus.

In addition to read mapping, we performed a survey of the NCLDV major capsid proteins (MCP) to test if the isolated and spiked-in viruses or other NCLDV were detectable in the metagenomic data. Identified metagenomic MCP were compared to sequences of MCP found in the Fadolivirus and Phoenician Marseillevirus reference genomes and to MCP available in the NCBI nonredundant (nr) database. Surprisingly, each sample only had between one and six MCP, most of which were on short contigs with low read coverage (Fig. 3b). These MCP showed only low sequence similarity to MCP of known NCLDV. Fadolivirus MCP were only detected in samples with the high- and medium-level of Fadolivirus spike-in, and Phoenician Marseillevirus MCP were not detected in any sample. In the metagenome from the sample with the highest level of Fadolivirus spike-in, all Fadolivirus MCP genes were correctly assembled and binned, whereas the samples with the medium level of Fadolivirus spike-in, Fadolivirus MCP genes, were present as short fragments distributed over 12 contigs in the unbinned fraction of the metagenome (Fig. 3b).

## DISCUSSION

Biological insights inferred from genomes extracted from metagenomes rely on sophisticated computational tools and algorithms designed to work efficiently and accurately on diverse sets of environmental sequence data. While these tools are applied on a daily basis by many biologists to answer ecological and evolutionary questions from uncultivated taxa of interest, benchmarking the results often falls short, with the exception of efforts such as Critical Assessment of Metagenome Interpretation (CAMI) (35) and the use of internal standards, such as spike-ins, in some studies (36, 37). This is in part due to the difficulty of performing such evaluations using a controlled experiment in a broadly applicable manner. To evaluate the performance of metagenomic assembly and binning of giant viruses, we conducted a benchmark experiment, where we spiked particles of a known giant virus, Fadolivirus, into a wastewater sample. Commonly used assembly and binning tools yielded a MAG which represented 98.3 to 99.7% of the Fadolivirus reference genome (Table 1). Of note, depending on the binning approach, contigs with a combined length of 15 kb (MetaBAT 2, without differential coverage binning) to 351 kb (CONCOCT) were wrongly assigned to the Fadolivirus MAG. This so-called “misbinning” is clearly a limitation of the metagenomic approach (38) and demands careful downstream evaluation based on other criteria, such as gene content (39). In contrast to the Fadolivirus reference genome assembly, our metagenomic workflow did not yield a closed genome. The presence of assembly breakpoints at highly conserved 80- to 200-bp repeats demonstrates the difficulty of using the metaSPAdes assembler (28) to resolve such repeats with shorter NovaSeq reads (2 × 150 bp, average insert size of 241 bp) compared to the longer reads (2 × 300 bp, average insert size of 253 bp) used for the reference assembly. The performance of the assembler was likely further reduced by the higher complexity of the wastewater sample, which contained more than 100 additional microbial MAG. However, compared to the microbial MAG, the Fadolivirus MAG had the highest *N*_50_ value and contained the largest contig. The Fadolivirus MAG did not have any misassembled regions and had a low mismatch rate of 16 nucleotides per 100 kb, which would correspond to a level of accuracy exceeding 99.98%. This comparably high quality of the metagenomic Fadolivirus assembly is likely due to the genomic homogeneity of the clonal Fadolivirus particles that were spiked in. Although this scenario is unlikely to reflect the average environmental sample, our results nicely demonstrate that metagenomics is a powerful tool to recover the nearly complete genome of a giant virus.

Some important aspects for environmental genomics of NCLDV need to be considered. Despite the high level of completeness of the Fadolivirus MAG compared to the viral reference, our pipeline classified it as being of archaeal origin and of low quality. While this may be expected, as we used a workflow which relies on a taxonomic framework established for bacterial and archaeal genomes, it also reveals a potential pitfall of metagenome projects. We assessed contamination, completeness, and taxonomy with the commonly used tool CheckM (30). Building on the CheckM output, MAG quality was then defined according to the MIMAG standards (31). The lack of most universal cellular marker genes in giant viruses prevents a correct completeness estimate and resulted in the Fadolivirus classification as a “low-quality” MAG. The taxonomic classification as archaea can be explained by the fact that the few marker genes which were present in the Fadolivirus genome were most similar to their eukaryotic homologs. The misclassification arises due to the absence of eukaryotic sequences in the CheckM reference database (30). Importantly, in giant virus metagenomics, misclassification is a known problem, as giant viruses have been deposited as either part of eukaryotic genomes or as bacteria (40, 41). In addition, integration of giant virus genes into host genomes cannot be excluded (42). Systematically evaluating the performance of our microbial MAG classification workflow on 230 published genomes of large and giant viruses, we found that 70% of them would have been classified as “Archaea” and all of them as “low quality” (data not shown). Thus, it has to be considered that some novel archaeal MAG in public databases might, in fact, be misclassified giant viruses. If MIMAG standards (31) are applied, successfully assembled and binned giant virus MAG should be recoverable from the low-quality MAG fraction.

Our results show that while a routinely used metagenomics pipeline would likely misclassify and/or not detect giant viruses, a targeted screening would potentially enable the recovery of nearly complete viral genomes. The quality of the resulting MAG could then be further assessed according to the Minimum Information of an Uncultivated Viral Genome (MIUViG) recommendations (7). However, a sample must have a sufficient abundance of giant virus particles for successful recovery and assembly of MAG, as illustrated by Fadolivirus, which assembled at 165× sequence coverage, but not at 2.4× (Fig. 3a), and by the Phoenician Marseillevirus, which could not be detected at all in the samples it was isolated from. Viruses may naturally be this abundant and clonal after viral replication in eukaryotic hosts. Importantly, sufficient abundances of giant viruses are common in environmental samples, as demonstrated in two recent studies in which hundreds to thousands of novel NCLDV genomes were successfully recovered from various metagenomic data sets (23, 43). In contrast, viral population heterogeneity or low abundance would complicate genome recovery. Thus, for the recovery of low-abundance viruses from complex environmental samples, isolation of giant viruses by cocultivation with suitable hosts is a highly effective approach (44, 45).

Our analysis of the MCP shows that surprisingly few sequence traces of giant viruses can be found in the wastewater sample despite up to 18 Gb of sequence data generated for each sample and the ability to cocultivate two NCLDV in the laboratory. Only up to 6 MCP from NCLDV (other than Fadolivirus) could be detected in each sample. Our results underscore that extraction of NCLDV from metagenomes, even in an era of terabase-scale next-generation sequencing, is limited by many lower-abundance viruses being beyond the sequence detection level. This does, however, hint at the presence of a vast novelty of currently undetected giant viruses across Earth’s ecosystems.

In summary, while this study highlights some limitations and important considerations to the extraction of giant virus genomes from metagenomes, such as the need for sufficient sequence coverage and the risk of blindly relying on taxonomic classification tools, it for the first time benchmarks and illustrates the validity of genome-resolved metagenomes in recovering a nearly complete, nonchimeric quality giant virus genome from a complex sample. Such benchmark data are invaluable for strengthening current and future studies focused on the genomes of uncultivated giant viruses, which are indispensable for capturing the extent of giant virus phylogenetic diversity and for making inferences about their host interactions and ecology (23, 43).

## MATERIALS AND METHODS

### Sample preparation.

Samples were collected in September 2018 from sewage prior to wastewater treatment in Toulon, France (GPS localization: N 43.119; E 5.904). Approximately 1 liter of wastewater was transferred to a sterile bottle and then stored at 4°C for 1 month before downstream experiments were performed.

### Giant virus cocultivation.

First, 30 ml of the wastewater sample was stained overnight with SYBR Green I nucleic acid gel stain (Molecular Probes, Life Technologies, USA). The sample was then processed by flow cytometry for sorting using the BD FACSAria Fusion cell sorter cytometer (BD Biosciences). After determining 40 populations, sorting was performed in 96-well microplates as previously described (46). Cocultivations were then performed on the sorted samples using Acanthamoeba castellanii strain Neff and *Vermamoeba vermiformis* as cell hosts, with 10 microplates for each host. Plates were incubated at 32°C and monitored by high content screening for giant virus detection (47).

### Giant virus identification.

Wells showing potential infection were processed by flow cytometry and scanning electron microscopy (TM4000 Plus microscope; Hitachi High Technologies, Japan) for presumptive identification as previously described (44, 47). Virus identification was further validated with PCR and genome sequencing (48).

### Giant virus spike-in experiment.

In parallel and independently of the sample described above, we isolated a novel virus from an Algerian sewage sample (26) by using the same coculture procedure as that used with *Vermamoeba vermiformis* (26). This virus was named Fadolivirus, and we used its particles to artificially contaminate the sample collected from Toulon, France. The rationale for using this particular virus as the spike-in was its genome being (i) large, at 1.6 Mb, and (ii) absent from public databases. The latter was critical, as this experiment was a truly blind study in which the U.S. team did not know the identity of the spike-in so as to minimize bias for genomic analysis. Three concentrations of Fadolivirus were selected for the spike-in experiment as follows: each tube, containing 35 ml of the homogenate sample, contained either 10^3^ viral particles/ml (low spike-in), 10^5^ viral particles/ml (medium spike-in), or 10^7^ viral particles/ml (high spike-in). Another 35-ml tube of the sample served as a no-spike control. After this step, the 4 tubes were centrifuged using a JA-20 rotor at 43,000 × *g* for 1 h and 30 min in an Avanti j-26 XP centrifuge (Beckman, France). The pellets of the 4 tubes were preserved at –80°C before transport and metagenome sequencing and analysis.

Viral particles were quantified by flow cytometry. Data were acquired using log scales for instrument scatter parameters and side scatter (SSC) and were associated with DNA content detected by the fluorescein (FITC) parameter after SYBR green staining as previously described (49). Thresholds were adjusted on the SSC parameter, and 10,000 events per sample were acquired. Acquisition and analysis were performed using BD FACSDiva Software and FlowJo. The quantification was performed using counting beads (Cytocount DakoCytomation, a suspension of concentration-calibrated fluorescent microspheres). The absolute count of the population was obtained using the following equation (50): (number of cells counted/number of Cytocount beads counted) × (Cytocount concentration; i.e., 1,054 beads/μl) × dilution factor.

### DNA extraction.

Metagenomic DNA from each of the four samples (no spike-in; low spike-in, 10^3^ viral particles/ml; medium spike-in, 10^5^ viral particles/ml; and high spike-in, 10^7^ viral particles/ml) was extracted using the DNeasy PowerSoil kit (Qiagen, Germantown, MD). As the samples were liquid, the manufacturer’s protocol was adjusted as follows: briefly, 35 ml of wastewater samples was centrifuged for 45 min at 10,000 rpm at 4°C. The supernatant was decanted, and the resulting pellet was resuspended in 500 μl of reserved supernatant. The resuspended pellet was then deposited in the kit’s bead tube in place of soil. The manufacturer’s protocol was followed thereafter. All DNA extracts were quantified using the PicoGreen assay and the Qubit 2.0 fluorometer (Invitrogen, Carlsbad, CA).

### Library creation and sequencing.

Sequencing libraries were created using the TruSeq DNA PCR-free DNA sample preparation kit following the manufacturer’s protocol (Illumina, San Diego, CA). Libraries were sequenced on the Illumina NovaSeq platform (2 × 150 bp) at the U.S. Department of Energy’s Joint Genome Institute (JGI), yielding between 14 and 18 Gb of sequence per sample.

### Metagenome assembly and binning.

Reads were corrected using bbcms 38.34 (http://bbtools.jgi.doe.gov) with the following command line options: bbcms.sh metadatafile=counts.metadata.json mincount=2 highcountfraction=0.6 in=out.fastq.gz out=input.corr.fastq.gz. The read set was assembled using the metaSPAdes assembler with metaSPAdes 3.13.0 (28). This was run using the following command line options: spades.py -m 2000 --tmp-dir scratch -o spades3 --only-assembler -k 33,55,77,99,127 --meta -t 72 -1 reads1.fasta -2 reads2.fasta.

The input read set was mapped to the final assembly, and coverage information was generated with bbmap 38.34 (http://bbtools.jgi.doe.gov). This was run using the following command line options: bbmap.sh nodisk=true interleaved=true ambiguous=random in=out.fastq.gz ref=assembly.contigs.fasta out=pairedMapped.bam covstats=covstats.txt bamscript=to_bam.sh.

Gene calling was performed with prodigal (51) using the -meta option. Contigs were organized into genome bins based on tetranucleotide sequence composition with MetaBAT 2 (29). Furthermore, we performed metagenomic binning with CONCOCT 1.1 (33), MaxBin 2.2.7 (32), and DAS_Tool 1.1.2 (34) with default settings on the assembly derived from the sample with the high virus spike-in.

### Identification of the Fadolivirus MAG.

To make this a blind study preventing any bias in the sequence data processing and analysis, the Fadolivirus reference sequence and any information about this isolate was kept in the LaScola laboratory until the viral MAG data were generated and analyzed at the JGI. The data were then revealed and compared. Diamond blastp was used to compare metagenomic proteins against the Fadolivirus reference genome (52). Only one MAG contained the proteins found in the Fadolivirus reference genome, which was used for detailed comparison with the Fadolivirus reference genome using QUAST (53), nucmer from the MUMmer package (54), and Circos to generate a whole-genome synteny plot (55).

### Survey of the major capsid protein.

A set of hidden Markov models (HMMs) for the NCLDV MCP gene was used in hmmsearch 3.1b2 (hmmer.org) with a cutoff of 1e-10 to identify putative MCP genes on metagenomic contigs. The resulting protein hits were extracted from the metagenome and subjected to diamond blastp (52) against the nr database (May 2019) and against all proteins found in the Fadolivirus reference genome.

### Data availability.

The Fadolivirus genome has been deposited at NCBI GenBank (accession no. MT418680 and MT418681). Metagenomic data sets can be retrieved from the IMG/M (18) database (no spike-in, IMG/M id 3300036762; low spike-in, 3300036763; medium spike-in, 3300036764; high spike-in, 3300036765) and the JGI genome portal (no spike-in, data set no. 1242206; low spike-in, 1242207; medium spike-in, 1242208; high spike-in, 1242209) and can also be directly downloaded from https://doi.org/10.6084/m9.figshare.12235733.v1.
